# Diagnostic Utility of Pre-Genomic Hepatitis B RNA in the Evaluation of HBV/HIV Coinfection

**DOI:** 10.20411/pai.v9i2.720

**Published:** 2024-07-30

**Authors:** Kenneth E. Sherman, Susan D. Rouster, Heidi Meeds, Marion G. Peters, Jason T. Blackard, Paul S. Horn, Timothy Archampong, Awewura Kwara, Mark Anderson, Michael Stec, Gavin A. Cloherty

**Affiliations:** 1 Division of Digestive Diseases, University of Cincinnati College of Medicine, Cincinnati, OH; 2 Department of Medicine, Northwestern University, Chicago, IL; 3 Department of Pediatrics, University of Cincinnati; Neurology Division, Cincinnati Children's Medical Center, Cincinnati, OH; 4 Department of Medicine and Therapeutics, University of Ghana Medical School, Accra, Ghana; 5 University of Florida College of Medicine, Gainesville, FL; 6 Abbott Laboratories, Abbott Diagnostics Division, Abbott Park, IL

**Keywords:** pgRNA, quantitative HBsAg, HBV DNA, HBV, HIV, treatment

## Abstract

**Background:**

Newer biomarkers of Hepatitis B virus (HBV) infection and treatment response have not been well-characterized in individuals with HBV/HIV coinfection.

**Methods:**

Pre-genomic RNA (pgRNA) and quantitative HBsAg (qHBsAg) were used to evaluate the associations with baseline characteristics. Participants included two separate groups – 236 with HBV/HIV coinfection enrolled in a cross-sectional cohort in Ghana and 47 from an HBV nucleoside/nucleotide treatment trial comparing tenofovir to adefovir in the United States.

**Results:**

In both cohorts, HBe antigenemia was highly associated with pgRNA and HBV DNA levels. In the treatment cohort, pre-treatment pgRNA serum concentration was 7.0 log_10_ U/mL, and mean qHBsAg was 201,297 IU/mL. The observed treatment-associated decrease in pgRNA was consistent with a biphasic decline curve that reached second-phase kinetics following treatment week 12. Changes from baseline were significantly correlated with changes in serum ALT (r = − 0.518; *P* = 0.023) but not with changes in HBV DNA (r = 0.132, *P* = NS). qHBsAg also correlated with ALT change (r = − 0.488, *P* = 0.034).

**Conclusion:**

pgRNA and qHBsAg represent newer biomarkers of HBV replication that may help monitor response and treatment outcomes. HBV pgRNA is highly associated with both HBeAg and ALT and may predict both active replication from the closed circular DNA (cccDNA) template as well as hepatic injury.

## INTRODUCTION

Hepatitis B virus (HBV) infection is common in individuals with HIV infection due to shared routes of transmission. Many epidemiological studies suggest that the rates of chronic HBV infection in individuals living with HIV (PLWH) range from 5% to 10% [1, 2]. Hepatic injury associated with HBV infection is common and manifests as an elevation of serum transaminases and the development of hepatic fibrosis. Long-term infection may lead to cirrhosis as well as an increased risk of hepatocellular carcinoma (HCC). Key markers that define the stage of infection include qualitative serum hepatitis B surface antigen (HBsAg), hepatitis B “e” antigen (HBeAg), antibody to HBeAg (anti-HBe), and serum HBV DNA titers. Serum ALT levels reflect the level of hepatic injury, and taken together, these markers define the current criteria used for treatment decisions and the evaluation of response [3]. More recently, attention has focused on the development of “functional cure,” which is broadly defined as the clearance of HBsAg from serum using a sensitive assay [4, 5]. A sensitive assay is defined as one whose lower limit of detection is 0.05 IU/mL while ultrasensitive assays may detect HBsAg at levels that are 10 to 100 times lower [6]. The need for a linkage between the appearance of anti-HBs antibody (HBsAb) and the subsequent clearance of HBsAg remains a subject of debate [7].

To best prognosticate treatment response outcomes as well as to guide the choice and sequence of future therapeutic interventions, novel biomarkers are needed to identify individuals who are most likely to achieve functional cure. To this end, there is an increased interest in the use of HBV pre-genomic RNA (pgRNA), an intermediary RNA product created by viral transcription of HBV covalently closed circular DNA (cccDNA) in infected hepatocytes, and quantitative HBsAg (qHBsAg), which measures the level of cccDNA activity. Direct detection of intrahepatic HBV cccDNA requires liver tissue obtained through biopsy, which may not always be available. Lin et al reported the use of serum pgRNA as a potential biomarker to quantify replication of the cccDNA template and correlated these levels with HBV DNA and HBsAg levels in patients with HBV infection receiving nucleos(t)ide analog therapy [8].

However, there are limited data regarding the use of these markers in PLWH. A recent study in China reported that HBV pgRNA was detected in only 34.4% of HBsAg-positive HBV/HIV individuals and was associated with HBeAg status and moderately correlated with HBV DNA titers. They suggested that loss of HBV pgRNA was possibly associated with HBeAg loss, though such clearance was only noted in 13 individuals [9]. In a review discussing the role of HBsAg quantitation in PLWH, Jakharia et al note that data regarding the use of this marker to prognosticate natural history is also limited [10]. Studies suggest that a one-log drop in HBsAg following one year of treatment with a nucleoside/nucleotide regimen had good to moderate sensitivity and specificity for eventual functional cure in those with low (<100 IU/mL) baseline HBsAg levels [11, 12]. A multicenter, prospective cohort study conducted in the United States followed 95 HBV/HIV coinfected individuals for up to 192 weeks. Most subjects (98%) were receiving HBV-active therapy in the setting of combination antiretroviral treatment. Even with complete HBV DNA suppression, HBV RNA detection indicated persistent viral transcription from the cccDNA [13]. In a study of HBV/HIV coinfected patients being treated with tenofovir disoproxil fumarate (TDF), Zoutendijk et al found that decreasing quantitative HBsAg levels during treatment were associated with an improvement in CD4 cell count [14].

To expand our understanding of the potential role(s) for these novel biomarkers of HBV/HIV coinfection, we developed an in-house assay for pgRNA using quantitative real-time PCR amplification, which was then validated via comparison with a Research Use assay developed by the coauthors at Abbott Laboratories [15]. We then utilized these assays to assess HBV pgRNA in two cross-sectional cohorts of participants with HBV/HIV coinfection as described below.

## METHODS AND STUDY POPULATIONS

### Study Populations

We assessed these novel prognostic and therapeutic biomarkers in two discrete HBV/HIV cohorts. The first cohort utilized samples from the AIDS Clinical Trials Group ACTG A5127, which was a US-based randomized, multicenter treatment trial of HBV in PLWH (NIH Clinical Trials Registration number: NCT00033163, registration date April 9, 2002). Participants were assigned (1:1) to receive either tenofovir (300 mg/day) or adefovir (10 mg/day) for 48 weeks for HBV suppression. Study criteria included HIV-1 infection with a stable antiretroviral treatment for at least 12 continuous weeks. Exclusion criteria included other liver diseases such as hepatitis C virus (HCV) or hepatitis D virus (HDV). The primary outcome was a change in serum HBV DNA from baseline to end-of-study, and the study was powered to achieve a non-inferiority endpoint. The majority (79%) of participants were HBeAg-positive, male, and 56% were White [16]. Serum samples were available in most participants from baseline, and weeks 4, 12, 24, and 48 of treatment.

The second study group consisted of a sub-Saharan cross-sectional Ghana cohort of HBV/HIV coinfected individuals who were seen between 2012 and 2014 at the Fever Unit of the Korle-Bu Medical School and Hospital in Accra, Ghana. The participants were either antiretroviral treatment-naïve or were on lamivudine (3TC) for at least 9 months and were enrolled to determine HBV viremia in treatment-naïve and treatment-experienced HBV/HIV coinfected patients and to identify the existence of HBV treatment resistance mutations [17, 18]. Most participants were African Black and female. Clinical information including demographics, HBV DNA levels, and other relevant laboratory studies was available for both cohorts. All samples were stored at −80^oC^ to maintain viral integrity. All participants in both cohorts were enrolled in IRB-approved protocols and informed consent was obtained at the time of entry into the study or registry. Samples and patient data were de-identified prior to the current analyses, and this study was adjudicated to be “not human subjects research” by the University of Cincinnati IRB.

### Pre-genomic HBV RNA Assays

Two assay methodologies were evaluated and utilized. A proprietary manual quantitative real-time PCR assay was developed at the University of Cincinnati lab. Briefly, this assay amplified a 170 bp transcriptional precore/core product of HBV cccDNA. Total viral RNA was extracted from patient serum/plasma using a QIAamp Ultrasens extraction kit (Qiagen), followed by qRT-PCR performed on a qPCR instrument (BioRad CFX96). An RT primer targeting the polyA tail of pgRNA (M-T15 [5′ – GAC CRA TCC TGT CAC CTC TGA CTT TTT TTT TTT TTT T – 3′)] was used to generate cDNA, followed by amplification with pgRNA-specific primers and a FAM-labeled probe (forward [5′ – GCA ACT TTT TCA CCT CTG CCT – 3′], reverse [5′ – GAC CRA TCC TGT CAC CTC TGA C – 3′], and probe [5′ – FAM-TTC AAG CCT CCA AGC TGT GCC TTG G-BQH – 3′]) [19]. Control conditions included no-RT reactions to confirm that only pgRNA was measured. A WHO standard was used for quantitation and the quantitative lower limit of detection was 3.02 log_10_ U/mL. This methodology was utilized for the Ghana cohort. The alternative assay performed by the Abbott Laboratories diagnostic development group was utilized for the ACTG treatment cohort and involved the performance of RNA-selective extraction chemistry followed by multiplex RT-qPCR amplifying target sites on the HBV X and core targets on the cccDNA using the m2000 automated system (Abbott Molecular). Initial studies involved a first-generation assay, which was modified to improve detection sensitivity in the second generation (HBV RNA 2.0) yielding a lower limit of quantitation (LLOQ) of 0.49 log_10_ U/mL (10 copies/mL), with version 1 and 2 results indistinguishable [20]. Prior analysis has established that HBV pgRNA is a stable biomarker in clinical samples that have been properly stored [20, 21].

Prior to applying pgRNA test methods to the two primary sample cohorts, we performed paired correlation of the two pgRNA methodologies, utilizing a set of 20 samples available from the Hepatitis Research Group repository at the University of Cincinnati. This registry is an ongoing longitudinal collection of participants seen in healthcare settings at the medical center and includes individuals infected with HBV with or without HIV or HCV. These samples were collected from 16 male and 4 female patients with a mean age of 45.3 years and included 2 HBV/HCV, 5 HBV/HIV, and 13 HBV-alone infections. The mean HBV viral load was 4.62 log_10_ IU/mL, with a mean ALT level of 142 U/L. Among those with HIV, HIV viral load was suppressed to below the level of quantitation.

### Quantitative HBsAg

qHBsAg titers in the Ghana cohort serum samples were determined using an enzyme-linked immunosorbent assay (ELISA) (Alpha Diagnostic International). Standards, controls, and samples were added to a pre-coated plate and incubated. The plate was washed and then enzyme conjugate was added and incubated. After a second wash, the substrate TMB was added and incubated again. Stop solution was added, and absorbance was measured at 450nm using a BioTek EPOCH plate reader. A standard linearized regression line was used to determine qHBsAg with a range of 1-20 ng/mL. Samples were run in multiple dilutions ranging from 1- to 5,000-fold until the sample was in the quantitative range of the assay. The ARCHITECT system with a LLOQ of 0.05 IU/mL was utilized to determine qHBsAg levels in the ACTG A5127 longitudinal treatment cohort. To facilitate a comparison between the two methodologies, HBsAg concentrations measured in ng/mL were converted to IU/mL using the conversion factor of 0.88 ng/1 IU as reported by Chudy et al [22].

### Statistical Methods

Data were analyzed as indicated using both parametric and non-parametric methods, as appropriate for the data type and distribution. Statistix 10.0 and SAS^(R)^ version 9.4 (SAS Institute Inc.) were employed in the analyses. A repeated measures model was utilized to examine HBsAg levels as a function of pgRNA and treatment week. To assess HBV pgRNA response levels following HBV treatment, a decline curve analysis was used to determine multi-phase kinetics.

## RESULTS

### Cohort Demographics and Other Characteristics

In the Ghana cohort with HBV/HIV coinfection, key characteristics were available for 236. The mean age was 40.9 years (range: 21–78 years). The majority were female (59.3%), and 92.8% of the entire cohort were African Black. The median CD4 count was 436 cells/mm^3^ and 65% of patients were being treated with antiretroviral therapy for HIV infection. The majority of treatment regimens identified included 3TC (lamivudine) [17, 18]. HIV viral loads were suppressed to below the limit of quantitation. In terms of HBV infection, HBeAg was present in 20% of cases while the remainder were anti-HBe positive.

The ACTG A5127 primary treatment cohort consisted of 47 participants with baseline samples available for analysis. This group was 94% male, and 55% were White. Baseline data from the two primary study cohorts are compared in Table 1.

**Table 1.** Study Cohort Demographics and Baseline Data

Characteristic	Ghana Cohort	ACTG Cohort
N	236	47
Mean Age (Range)	40.9 (21 – 78)	43.6 (32 – 63)
Median CD4 cell count / mm^3^ (Range)	436 (2 – 1781)	458 (110 – 984)
Sex	Male 40.7%; Female 59.3%	Male 93.6%; Female 6.4%
Race/Ethnicity	African Black 92.8%; Unknown 7.2%	White, non-Hispanic 55.3%; Black, non-Hispanic 34%; Other 10.7%
ALT Mean (Range)	35.2 U/L (10 – 155)	64.9 U/L (11 – 365)
HBeAg Positive	19.9%	79%
HBV DNA, Mean log_10_ IU/mL + S.D. HBV DNA, Median log_10_ IU/mL	3.3 + 2.7 2.04	8.35 + 0.85 8.85
qHBsAg, Mean IU/mL + S.D. (range) qHBsAg, Median, IU/mL	9313 + 19677 (1 – 130077) 2632	201297 + 274726 (1 – 1228914) 117881
Treatment with 3TC, (%)	65	94

### HBV pgRNA Assay Calibration and Concordance

**Figure 1.**
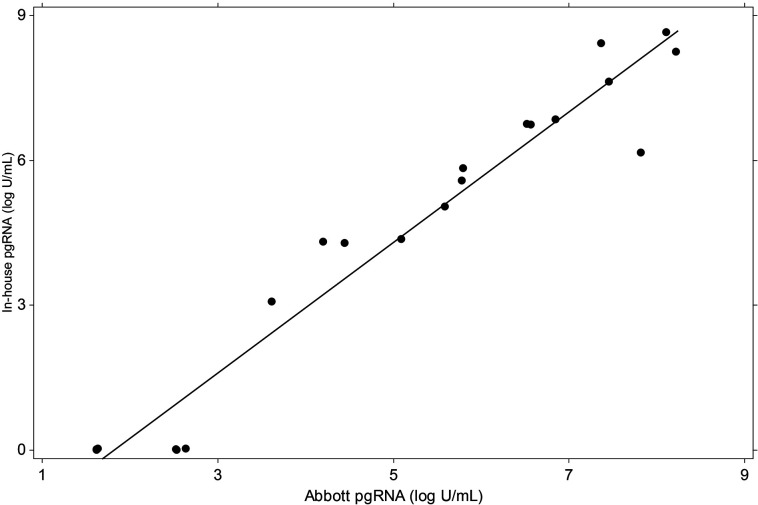
Correlation of the pgRNA methods utilized.

Calibration of the two HBV pgRNA assays was performed using extracted HBV DNA secondary standards traceable to the WHO HBV DNA standard. The linearity of these assays was evaluated in triplicate on 10-fold dilution series panels prepared from an HBV pgRNA-positive patient [15]. Figure 1 (below) demonstrates that the two assays are highly concordant (r = 0.973; *P* < 0.0001).

### Baseline Levels and Relationships between HBV Biomarkers

**Figure 2.**
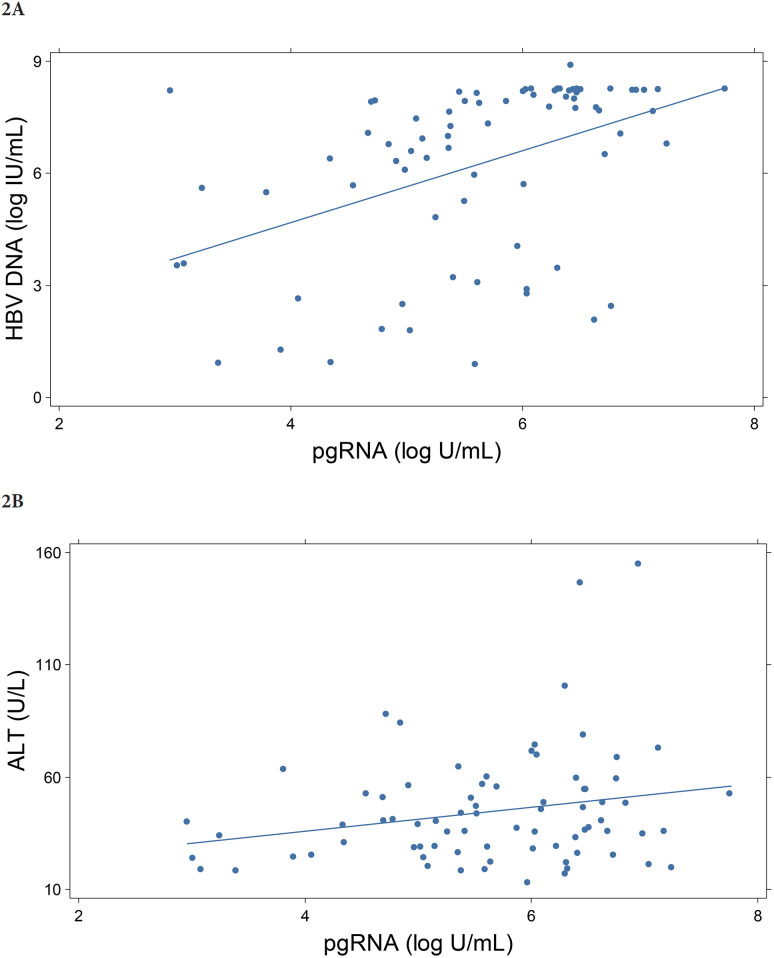
**Graphs showing pgRNA relationships in the Ghana cohort.** 2A depicts the association of log_10_ HBV DNA and pgRNA, (r = 0.44; *P* = 0.0001); 2B illustrates ALT and pgRNA correlation, (r = 0.23; *P* <0.05).

In the Ghana cohort, the mean HBV DNA viral load was 3.3 log_10_ IU/mL, and the pgRNA titer was 1.9 log_10_ U/mL. HBV pgRNA was detectable in only 33.8% of the cohort. Among those with detectable pgRNA, log_10_ HBV DNA levels were significantly correlated (r = 0.44; *P* = 0.0001) (Figure 2A). The mean qHBsAg titer was 9313 (range: 1-130,077) IU/mL. HBe antigenemia was highly associated with both pgRNA and HBV DNA levels (*P* < 0.001). Among HBeAg-positive participants, the mean level of pgRNA was 5.85 vs 0.98 log_10_ U/mL for HBeAg negative persons. A similar relationship was observed between HBeAg and qHBsAg, with much higher levels among those with HBe antigenemia (29,345 vs. 4,413 IU/mL; *P* < 0.001).

In the ACTG treatment cohort, the mean baseline HBV DNA was 8.35 (+0.85) log_10_ IU/mL. Pre-treatment pgRNA serum concentration was 7.0 (+1.3) log_10_ U/mL, and mean qHBsAg was 201,297 + 274,726 IU/mL. Overall, 81.8% of participants had detectable HBV pgRNA. The relationship with HBe status was similar. Levels of pgRNA, HBV DNA and qHBsAg were all higher among HBeAg positive persons.

### Decline of HBV pgRNA with Nucleotide Treatment

**Figure 3.**
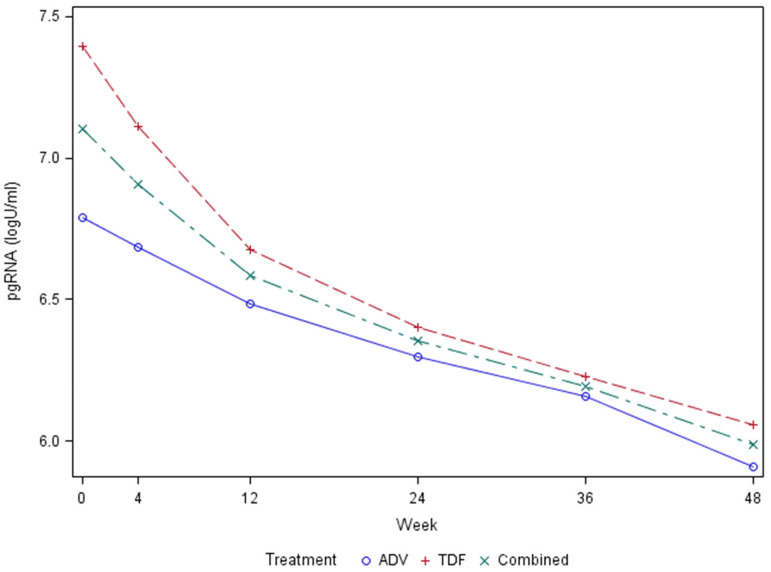
Graph showing pgRNA biphasic decline curve after HBV treatment in the ACTG cohort.

In the ACTG A5127 treatment trial, sample collection across several timepoints allowed for the assessment of HBV pgRNA decline curves. Figure 3 shows the decline curves for all subjects, as well as for treatment with adefovir versus tenofovir disoproxil fumarate. These results are consistent with a biphasic decline curve that reaches second-phase kinetics following week 12 of treatment. Following initiation of treatment, pgRNA declined more in individuals treated with TDF versus ADV at weeks 12 (0.24 log_10_ U/mL) and 48 (0.30 log_10_ U/mL), although this failed to reach statistical significance. Regression analysis showed that the rate of decline was significantly associated with age, race, treatment week, baseline ALT level, and baseline HBV DNA, (*P* < 0.05). Only 3 participants cleared HBV pgRNA relative to baseline levels.

There appears to be a relationship between pgRNA and qHBsAg. Specifically, in a repeated measures model where qHBsAg is modeled as a function of pgRNA and treatment week, then the coefficient is statistically significant for pgRNA on log-scale with AR (1) (first-order autoregressive) covariance structure, with the omission of one outlier. The resultant slope = 0.59, S.E. = 0.09.

### Relationship Between HBV Biomarkers and Liver Injury

**Figure 4.**
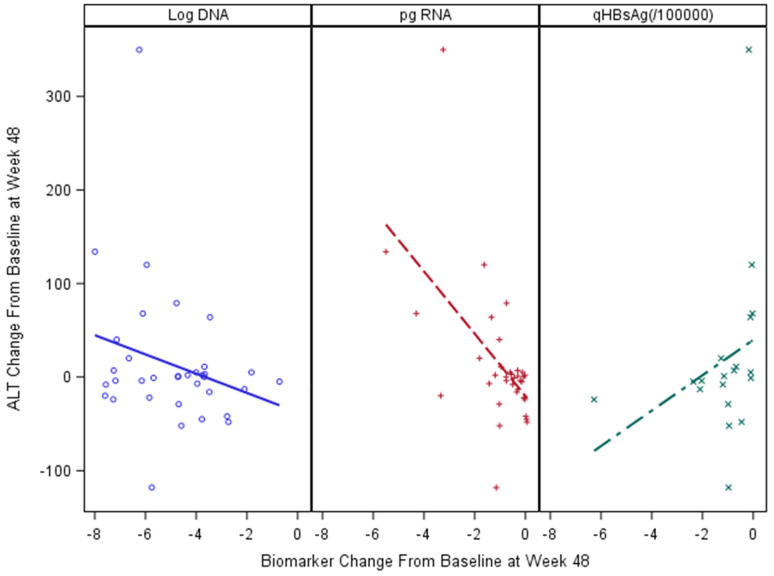
Changes in biomarker levels of active liver injury and relationship to ALT change from baseline to treatment week 48 in the ACTG cohort.

Serum ALT levels are a key marker of active liver injury. We examined the relationship of ALT to HBV biomarkers in baseline samples. Among 227 participants in the Ghana cohort, HBV pgRNA was most strongly associated with serum ALT levels (r = 0.23; *P* < 0.05), and log_10_ HBV DNA viral load (r = 0.44; *P* < 0.0001), (Figure 2A and 2B). In univariate comparisons, age and sex at birth were not associated with pgRNA levels or serum ALT, (*P* > 0.05). In the longitudinal treatment cohort, changes from baseline were significantly correlated with change in serum ALT (r = − 0.518; *P* = 0.023), but not with the change in HBV DNA (r = 0.132, *P* = NS). qHBsAg was also correlated with ALT change (r = − 0.488, *P* = 0.034), (Figure 4).

## DISCUSSION

For more than two decades, the goal of therapeutic intervention for HBV has been to suppress viral replication with the hope of achieving seroconversion among HBeAg-positive persons to reduce inflammation, slow hepatic fibrosis progression, and lower the risk of HCC development in both HBeAg-positive and -negative persons. In recent years, there has been renewed interest in achieving “functional cure,” with loss of HBsAg with or without the appearance of anti-HBs. Multiple drug targets used singly or in combination are being explored [23–25]. However, the change in the goals of therapy highlights the need for new diagnostic and prognostic tools that can identify which subpopulations are more or less likely to respond to a given regimen and to provide guidance for futility once treatment has been initiated. Recent interest has focused on the use of qHBsAg levels, for which lower levels (<100–300 IU/mL) are more likely to be associated with ultimate HBsAg clearance. However, the relatively low frequency of functional cure events associated with current therapies has made this predictor difficult to study. HBV pgRNA is another marker with the potential to better characterize the replicative status of individual patients. Its reflection of the transcriptional activity of the cccDNA template offers opportunities to better understand both replicative biology and therapeutic response. First, we cross-validated two different methodologies for detection and quantitation of HBV pgRNA. These were found to be highly correlated which allowed us to directly compare two discrete HBV/HIV cohorts with respect to demographic, serologic, and virologic characteristics. The inclusion of these two distinct cohorts allowed us to validate the findings across a broader spectrum of HBV/HIV coinfection.

Neither diagnostic assay – qHBsAg or HBV pgRNA – has been characterized in large cohorts of patients with HBV/HIV coinfection, which represent a unique but common subgroup whose immunobiology and disease course is altered by the bidirectional effects of each virus on the other. Jakharia and colleagues reviewed the role of qHBsAg among HBV/HIV coinfected persons [10]. They conclude that HBsAg levels tend to be approximately 1 log higher among PLWH with low CD4 counts or in those with advanced AIDS. As in patients with HBV monoinfection, those who are HBeAg positive have higher qHBsAg levels than those with antibody. Strassl et al suggested that a baseline level of qHBsAg <100 IU/mL in HBeAg-negative patients was 100% sensitive at predicting HBsAg loss with a specificity of 83% [26]. Data regarding HBV pgRNA are more limited. A cohort in China (n = 132) reported a strong association between HBV pgRNA levels and HBeAg status [9]. A similar association was described at baseline among 92 PLWH from 8 US centers [27]. This is similar to our findings in this report. In the Ghana cohort, the majority of participants were HBeAg negative as compared to the ACTG group, in which most were HBeAg positive. However, a strong linkage between HBe antigenemia and pgRNA titer was observed in both cohorts, supporting the concept that HBeAg-positive individuals are much more transcriptionally active than those without antigen. Indeed, the majority of participants in the Ghana cohort were HBV pgRNA negative. Unfortunately, initiation of nucleotide therapy in the ACTG cohort did not stop cccDNA transcriptional activity. Longer treatment duration may lead to pgRNA clearance; however, that could not be determined in this study cohort. Recent data from Balagopal et al using single-cell methods help explain this concept with HBeAg-negative individuals having less active transcription from cccDNA and greater levels of HBV gene integration into the host genome [28, 29].

Our observation that the HBV pgRNA decline curve is biphasic following treatment with tenofovir or adefovir is mirrored by similar observations among HBV monoinfected patients [30].

This observation may allow predictive modeling of clearance using more advanced models, which were highly informative of viral clearance of HCV in those with HCV/HIV coinfection [31]. Increased early sampling and application of dynamic models may further our understanding of responses related to single and combination therapies for HBV in the setting of HIV. While our cohorts did not permit evaluation of the role of pgRNA in predicting functional cure, a recent publication suggests that there may be utility in this determination [32].

The association between pgRNA and serum ALT levels is intriguing. Hepatic injury in HBV is thought to result from host immunologic clearance mechanisms. Higher serum pgRNA level may reflect either significant levels of viral replication from a limited number of hepatocytes or active replication occurring in a greater proportion of hepatocytes. Without liver biopsy correlation, it is not possible to say which of these states is present. Immune-mediated clearance of infected hepatocytes is thought to be primarily associated with highly replicative HBV, which historically has been determined by examination of HBV DNA level and presence of HBeAg. Since pgRNA reflects transcriptional activity, it may serve as a better marker of immune-mediated clearance than HBV DNA. Going forward, it will be important to determine whether transcriptionally silent cells that contain cccDNA can evade immune clearance mechanisms.

Limitations of our data include the relatively small treatment cohort and the lack of follow-up in that cohort beyond the initial 48 weeks of treatment. The cohorts utilized were enrolled before the use of tenofovir alafenamide (TAF), which is now largely replacing the use of TDF. It is not known if the Ghana cohort, which consists of African Black PLWH from sub-Saharan Africa, is representative of the distribution of HBeAg/HBeAb that might be observed in the US or elsewhere. Liver biopsies were not available in either cohort, along with the characterization of fibrosis; thus, analysis of cccDNA and correlation with other biomarkers was not possible. Neither Hepatitis Delta (HDV) nor HCV status were known for the Ghana cohort and could theoretically influence transcriptional activity. No information regarding body mass index, diet, alcohol consumption, sexual activity, or travel was available for either cohort. Although the two methods utilized for HBV pgRNA levels were highly concordant, the assay used for the ACTG treatment cohort had a lower limit of detection, which may have contributed to the higher detection rates seen in that cohort.

In conclusion, novel biomarkers are needed to link the prognosis and outcomes of hepatitis B treatment to new agent(s) capable of increasing HBsAg clearance leading to a functional cure. In the setting of HIV infection, it is particularly important to determine the diagnostic/prognostic characteristics of markers, such as pgRNA and qHBsAg, as they may differ from those with HBV monoinfection. Our data raises the possibility that pgRNA clearance may precede HBeAg to HBeAb conversion, but longer treatment trials that identify seroconversion are needed to validate this. Continued transcription from the cccDNA template implies ongoing active replication which may be directly targeted by newer experimental therapies (eg, siRNA). Thus, HBV pgRNA could help identify individuals who require personalized approaches to therapy.
